# Atlas of Travel Medicine and Health

**DOI:** 10.3201/eid0911.030503

**Published:** 2003-11

**Authors:** Lin H. Chen

**Affiliations:** * Mount Auburn Hospital, Cambridge, Massachusetts

Travel medicine is a field that requires in-depth knowledge about disease epidemiology. Whether providing pretravel evaluations or posttravel consultations, the travel medicine specialist must be up to date on disease epidemiology. Pretravel evaluations require counseling the traveler to avoid risks from diseases specific to the itinerary. Posttravel consultations demand recognition of possible diseases on the basis of areas visited as well as specific exposures. A pictorial depiction of disease distribution can be a useful tool in determining risks of diseases to the traveler and can complement the existing resources for clinicians.

Atlas of Travel Medicine and Health, a book and accompanying CD-ROM by J. Chiodini and L. Boyne, contains basic information that can be used to provide pretravel advice. Its major sources are the World Health Organization and U.K. Department of Health and the Public Health Laboratory Service guidelines. The book is organized into three sections. Section One describes general risks and precautions, such as food and water, animal contact, vector-borne diseases, fresh water exposure, and blood and body fluid contact. Section Two contains descriptions and maps of some diseases that may be encountered by travelers. Section Three contains country-specific information about malaria, immunizations, and other health considerations for the more popular destinations.

The format is organized, the content is basic, and the book is easy to read. Although the intended audience is healthcare professionals, the information can be easily used by the traveler. Some maps have incorporated the topography to indicate specific malaria risk, depending on elevation, which is a nice feature.

Because Section Three only focuses on popular destinations, many countries with malaria risk are not included. The section can be improved with consistent use of color, a more detailed key, and by adding major tourist centers and cities. Although the authors do not intend to cover an exhaustive list of diseases or countries, another important improvement would be the inclusion of all countries infected with or endemic for yellow fever. Diseases such as leishmaniasis, Lyme disease, and acute mountain sickness deserve mention in Section Two but are not currently discussed. Finally, the CD-ROM is generally easy to use, but Section Two on the CD-ROM could be improved by changing the orientation of the maps.

We live in a world of movement. The mobility of populations globally leads to the potential spread of infectious diseases and to the rapid change of disease distribution as demonstrated by outbreaks of dengue, meningococcal disease, and severe acute respiratory syndrome (SARS). An ideal atlas for travel medicine should be able to reflect recent outbreaks in addition to the general distribution of diseases. To do so would require frequent updates of the atlas. Currently, the Atlas of Travel Medicine and Health is suitable for healthcare professionals and travelers who desire brief summaries on some travel-related illnesses, but it must be used in conjunction with more comprehensive references.

